# Locating the depth of magma supply for volcanic eruptions, insights from Mt. Cameroon

**DOI:** 10.1038/srep33629

**Published:** 2016-10-07

**Authors:** Harri Geiger, Abigail K. Barker, Valentin R. Troll

**Affiliations:** 1 Centre for Experimental Mineralogy, Petrology and Geochemistry (CEMPEG), Department of Earth Sciences, Uppsala University, Villavägen 16, SE 752 36, Uppsala, Sweden; 2 University of Las Palmas de Gran Canaria, Department of Physics (GEOVOL), Las Palmas de Gran Canaria, Spain

## Abstract

Mt. Cameroon is one of the most active volcanoes in Africa and poses a possible threat to about half a million people in the area, yet knowledge of the volcano’s underlying magma supply system is sparse. To characterize Mt. Cameroon’s magma plumbing system, we employed mineral-melt equilibrium thermobarometry on the products of the volcano’s two most recent eruptions of 1999 and 2000. Our results suggest pre-eruptive magma storage between 20 and 39 km beneath Mt. Cameroon, which corresponds to the Moho level and below. Additionally, the 1999 eruption products reveal several shallow magma pockets between 3 and 12 km depth, which are not detected in the 2000 lavas. This implies that small-volume magma batches actively migrate through the plumbing system during repose intervals. Evolving and migrating magma parcels potentially cause temporary unrest and short-lived explosive outbursts, and may be remobilized during major eruptions that are fed from sub-Moho magma reservoirs.

Mt. Cameroon is one of Africa’s largest and most active volcanoes with eight major eruptions recorded in the last century alone^1^ (1909, 1922, 1925, 1954, 1959, 1982, 1999 and 2000; Fig. 1). Despite the imminent risk of volcanic eruptions, earthquakes and associated landslides, the flanks of Mt. Cameroon are densely populated by ≥0.5 million people^2^ who are drawn to the region by the fertile soil and the associated economic benefits. Effective monitoring, early warning systems and up-to-date emergency plans are scarce^3^, which is why further research into the internal workings of the volcano is critically needed to support informed hazard mitigation strategies.

Mt. Cameroon (4.20°N, 9.17°E; 4095 m asl) is located on the Atlantic coast of the Republic of Cameroon and forms part of the Cameroon Volcanic Line, a ~1600 km long chain of oceanic to continental volcanoes^4^,^5^,^6^,^7^,^8^. The Cameroon Volcanic Line stretches from the Atlantic island of Pagalu onto the African continent as far as Lake Chad and is comprised of twelve major volcanic centres^9^,^10^. Mt. Cameroon is a NE-SW aligned elliptical stratovolcano (50 km × 35 km; Fig. 1b) with a total volume of ~1200 km^3^ (ref. 2). It erupts dominantly basanite and alkali basalt through a basement of Precambrian metamorphic crust and Cretaceous to Quaternary sedimentary rocks^4^,^5^,^6^. The last two sustained eruptions at Mt. Cameroon occurred between 28 March and 22 April 1999 and between 28 May and 19 June 2000 (refs 5 and 8). Mt. Cameroon displays over 100 cones or vent sites on its flanks and in the summit region, which are typically also elongated in a NE-SW direction^8^,^11^. Seismic activity during times of eruptive quiescence defines background levels of up to one small earthquake (magnitude <3) per day, as well as the occurrence of frequent seismic swarms and tremors with a hypocentre distribution from close to the surface down to ~60 km depth^12^,^13^,^14^. Seismic activity generally increases prior to major eruptions^14^ and the 1999 and 2000 eruptions were preceded by increased seismicity between 20 and 55 km depth^8^. In addition to the two sustained volcanic eruptions of 1999 and 2000, short-lived volcanic explosions occurred in 1989 and 2012, i.e. within the quiet repose intervals. Neither of these led to lava emission and both lasted for seconds only^13^,^15^. Realizing that some seismic events lead to eruptions, while others do not, a clearer picture of the internal architecture and magmatic processes of Mt. Cameroon is required in order to provide a better understanding of the magma supply system that feeds Mt. Cameroon volcano and to mitigate volcanic risks in the region. We therefore employed mineral-melt equilibrium thermobarometry on the most recent lavas and their minerals in order to provide a first order reconstruction of the magma storage levels beneath the volcano.

## Results

### Petrography and geochemistry

**Figure 2:**
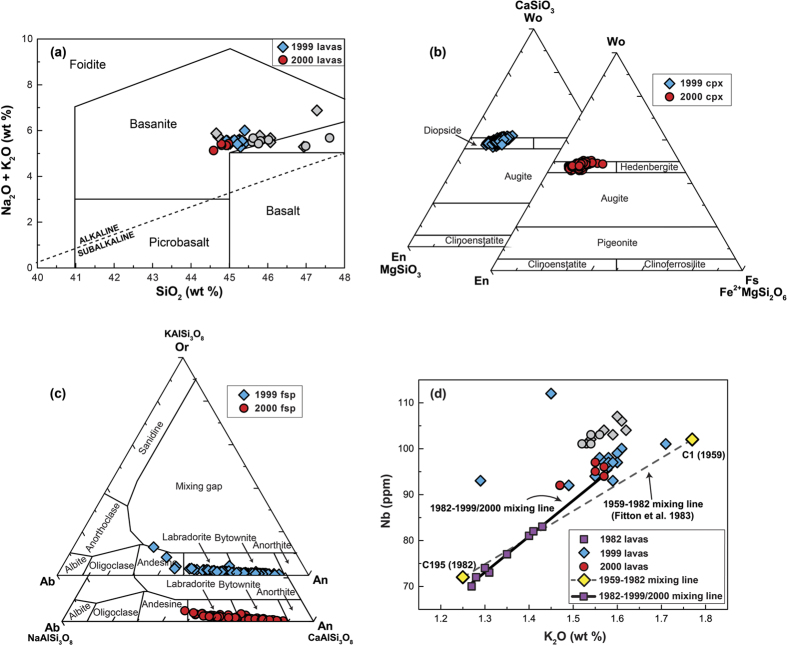
Geochemical data for Mt. Cameroon lavas and minerals. (**a**) Total alkali versus silica diagram after Le Bas *et al*.^49^ classifies the 1999 and 2000 Mt. Cameroon lavas as basanites. Previously published data^8^ are plotted as grey symbols. (**b**) Clinopyroxenes from the 1999 and 2000 eruptions (n = 527) classify as diopside, following the scheme of Morimoto *et al*.^50^. (**c**) Plagioclase compositions from the 1999 and 2000 eruptions (n = 364) classify dominantly as bytownite and labdradorite, with only minor andesine present. (**d**) Plot of Nb versus K_2_O for the 1982, 1999 and 2000 lavas. Mixing trends are observed between the 1959 and the 1982 lavas (Fitton *et al*.)^7^ and between the 1982 and the 1999/2000 lavas (this study). Previously published data on the 1999 and 2000 eruption^8^ are plotted as grey symbols.

Samples from the 1999 and the 2000 lavas are exclusively basanites, but the 1999 lavas are slightly more evolved relative to the 2000 ones (by ~1 wt% SiO_2_, Fig. 2a). The 1999 and 2000 lavas contain ~20 to 25% phenocrysts of euhedral to subhedral olivine, clinopyroxene and plagioclase set in a fine-grained, partly vesicular and microlite-bearing groundmass. Olivine phenocrysts make up ~10% of the samples and are usually ≤5 mm and unzoned. The compositional range of olivine is Fo_67–86_ with an average of Fo_78±10_ (2σ, n = 81). Clinopyroxene up to 3 mm in size also constitutes up to 10% of the 1999 and 2000 lavas and shows a compositional range of Wo_46–50_En_31–46_Fs_7–20_. Pyroxene classifies as diopside (Fig. 2b), with Mg numbers (Mg#) between 61 and 86 and an average Mg# of 77 ± 3 (2σ, n = 527). Normal and reverse zoning is recorded by Mg# in clinopyroxene from both eruptions. Phenocrysts of plagioclase feldspar (≤5%) are up to 1 mm in size and classify as andesine, labradorite and bytownite with a range of An_32–88_ and an average of An_72±9_ (2σ, n = 364; Fig. 2c). Variations in anorthite content of plagioclase from both eruptions record strong normal and reverse zoning. When compared with older Mt. Cameroon eruption products, the 1999 and 2000 lavas display higher Nb and K_2_O values than the 1982 lavas, but are close to the composition of the 1959 lavas. In fact, the 1999 and 2000 lavas fall onto a projected mixing line with the 1982 lavas (Fig. 2d), which is similar to but slightly offset from the previously identified 1959–1982 mixing line^7^.

### Clinopyroxene-melt thermobarometry

To provide insight into the depth of magma storage and the magmatic processes beneath Mt. Cameroon, we employed the clinopyroxene-liquid equilibrium thermobarometer of Putirka *et al*.^16^. This approach is widely used in recent petrological studies and delivers pressure results for a wide range of clinopyroxene-melt combinations that have been independently confirmed by a range of other methods^17^,^18^,^19^,^20^,^21^,^22^. The approach is a recalibration of the model of Putirka *et al*.^23^ and is based on the jadeite-diopside/hedenbergite exchange between clinopyroxene and the former melt composition. The 2003 version of the method also accounts for hydrous compositions (Table 4 in Putirka *et al*.; ref. 16) and the standard errors of estimate (SEE) for pressure and temperature determinations are ±33 °C and ±0.17 GPa, respectively^16^.

Other available thermobarometers, whether using clinopyroxene composition only or clinopyroxene-melt couples, are not suitable for Mt. Cameroon lavas. The geobarometer of Nimis^24^ employs single clinopyroxene compositions, however it is not calibrated for alkaline silica-undersaturated systems such as those at Mt. Cameroon (cf. ref. 25). A more recent approach by Putirka (ref. 26; Eq. 32c) is based on Al partitioning between clinopyroxene and melt. It requires H_2_O input, and is superior to previous hydrous models with respect to precision. However, this formulation is also not calibrated for alkaline systems and thus cannot be employed for the recent Mt. Cameroon lavas. Neither is the approach by Yang *et al*.^27^,^28^ suitable, which makes use of the olivine-plagioclase-clinopyroxene cotectic boundary. A new barometer by Masotta *et al*.^29^ is based on clinopyroxene-liquid equilibria and is calibrated for evolved alkaline magmas. This barometer produces results for Mt. Cameroon that are highly consistent with the ones we obtain using Putirka *et al*.^16^, however, the formulation of Masotta *et al*.^29^ is specifically calibrated for evolved magmas and not for mafic compositions, and therefore it cannot be readily applied to the mafic Mt. Cameroon lavas either.

**Figure 3:**
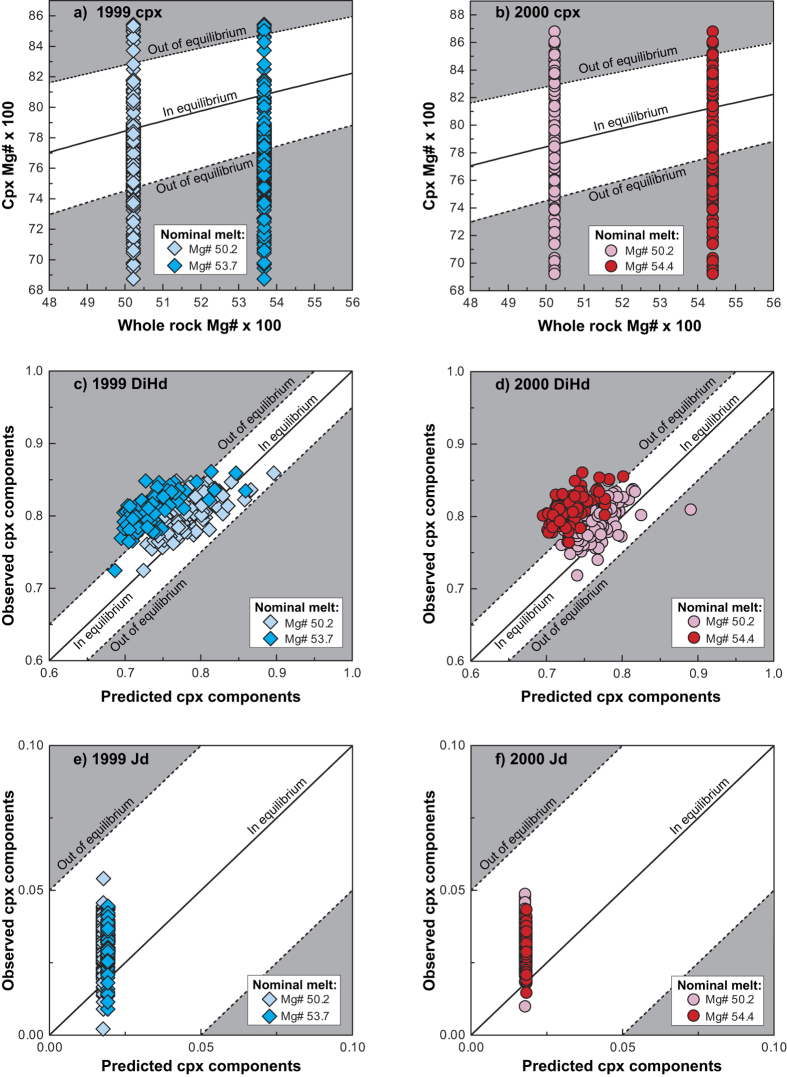
Equilibrium tests for clinopyroxene. Equilibrium test for (**a**) clinopyroxene from the 1999 eruption paired with whole rock data of Mg# 53.7 (ref. 8) and Mg# 50.2 (ref. 7). (**b**) Clinopyroxene from the 2000 eruption paired with whole rock data of Mg# 54.4 (ref. 8) and Mg# 50.2 (ref. 7). Both tests (**a**,**b**) employ a K_D[FeMg]_ of 0.275 ± 0.067 to define the equilibrium envelope^16^ and all data points that fall within this envelope are used for further equilibrium assessment. (**c**) Predicted versus observed clinopyroxene mineral components (diopside and hedenbergite) for the 1999 pyroxenes paired with the Mg# 53.7 and Mg# 50.2 nominal melts. (**d**) Predicted versus observed clinopyroxene mineral components (diopside and hedenbergite) for the 2000 pyroxenes paired with the Mg# 54.4 and Mg# 50.2 nominal melts. (**e**) Predicted versus observed clinopyroxene mineral components (jadeite) for the 1999 pyroxenes paired with the Mg# 53.7 and Mg# 50.2 nominal melts. (**f**) Predicted versus observed clinopyroxene mineral components (jadeite) for the 2000 pyroxenes paired with the Mg# 54.4 and Mg# 50.2 nominal melts. Only data that satisfy all equilibrium conditions (i.e. that fall within all tested equilibrium envelopes) are taken further for thermobarometry modelling.

To establish if the crystals in these recent lavas are in equilibrium with the available nominal melt compositions, the Mg# of clinopyroxene is plotted against the Mg# of the potential nominal melts. This equilibrium test is based on Fe/Mg partitioning, which makes it necessary to estimate the Fe^3+^/Fe_total_ ratio of the melt. Mt. Cameroon lavas are compositionally similar to ocean island basalts (OIB), which have Fe^3+^/Fe_total_ ratios of 0.15–0.25 (ref. 30). We thus assumed an intermediate Fe^3+^/Fe_total_ ratio of ~0.21 for Mt. Cameroon lavas, consistent with for example compositionally similar alkaline basanites from the Canary Islands^31^. Clinopyroxene Fe^3+^ content was assumed to be zero, consistent with the calibration of K_D[FeMg]_ in Putirka *et al*.^16^. To constrain the magma plumbing system of Mt. Cameroon, nominal melts were selected from whole rock compositions of the 1982, 1999 and 2000 eruptions (Mg# 50.2, Mg# 53.7 and Mg 54.4, respectively)^7^,^8^. We account for a large span of crystal compositions by employing a range of whole rock compositions from Mg# 50.2 to 54.4 that extend over the previously identified differentiation trends^7^. The use of melt inclusions as nominal melt was avoided due to their scarcity and potentially ambiguous representation of the original melt composition^32^. Similarly, groundmass glass analyses were not employed due to the high microcryst content of the groundmass, which will have modified the original melt composition during late stage solidification. Data points that then plot within the K_D[FeMg]_ = 0.275 ± 0.067 envelope represent crystals in equilibrium with the tested nominal melts (cf. ref. 16), which is the whole rock composition in this case (Fig. 3a,b). These data are considered further. In a next step, we evaluated the predicted versus observed clinopyroxene mineral components. This equilibrium test evaluates the conformity of predicted and observed diopside-hedenbergite (DiHd), enstatite-ferrosilite (EnFs), calcium-Tschermak (CaTs) and jadeite (Jd) clinopyroxene components. Data points that fall close to the one-to-one line (within ±10%) are assumed to validate equilibrium conditions and are used for thermobarometric calculations (Fig. 3c–f). Clinopyroxene crystals in equilibrium with the Mg# 53.7 nominal melt^8^ range in Mg# from 77 to 85. When clinopyroxene is coupled with the Mg# 54.4 nominal melt^8^, the equilibrium clinopyroxene range is Mg# 78 to 85. In turn, when coupled with the Mg# 50.2 nominal melt^7^, equilibrium crystals in the 1999 and 2000 eruptions range from 75 to 83 in Mg#. This range of equilibrium nominal melt compositions thus represents the compositional spectrum of the magmatic liquids (melts) that were involved during crystal growth.

**Figure 4:**
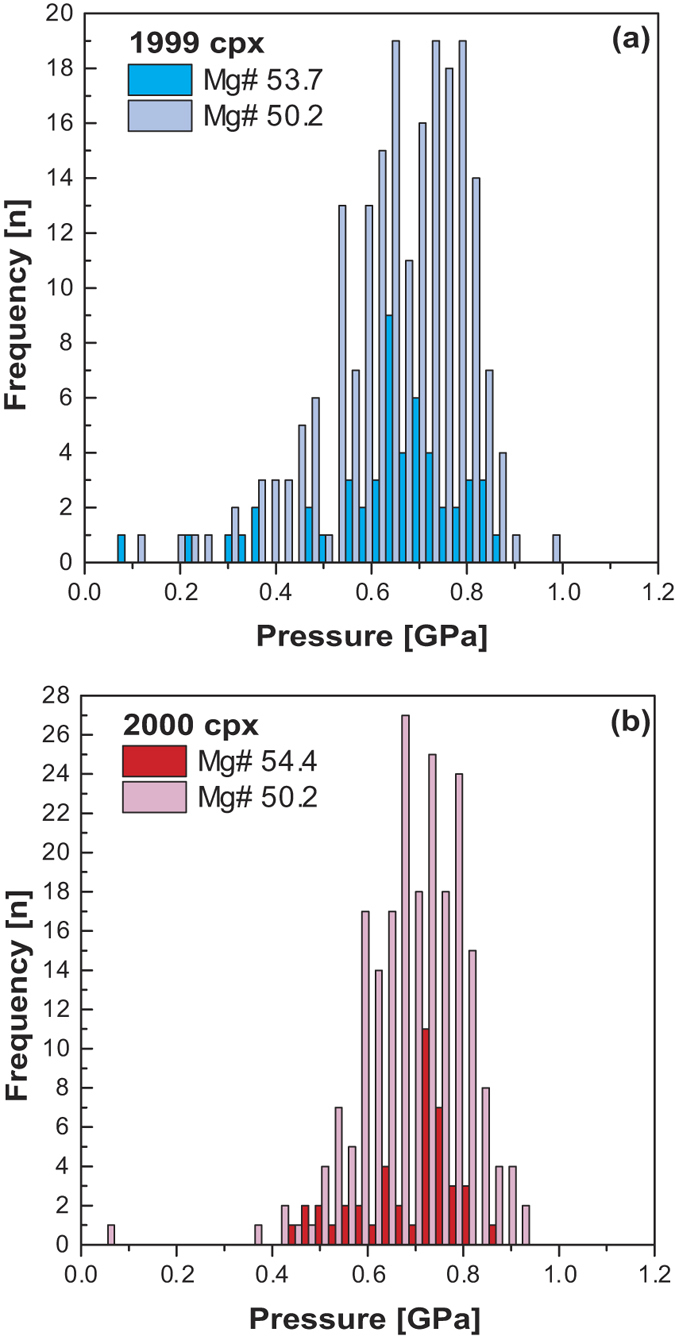
Clinopyroxene crystallization pressures for recent Mt. Cameroon lavas after Putirka *et al*.^16^. (**a**) Clinopyroxene from the 1999 lavas coupled with equilibrium melts of Mg# 53.7 or Mg# 50.2 (SEE = ±0.17 GPa). (**b**) Clinopyroxene from the 2000 lavas coupled with equilibrium melts of Mg# 54.4 or Mg# 50.2 (SEE = ±0.17 GPa).

In order to convert the obtained pressure data to depth values, a density of 2.8 g/cm^3^ was employed for the basaltic lithosphere beneath the volcano. The 1999 and 2000 eruption products yield crystallization pressures that range from 0.08 to 0.97 GPa for the 1999 lavas (melt Mg# 53.7; Fig. 4a) and from 0.35 to 0.97 GPa for the 2000 lavas (melt Mg# 54.4; Fig. 4b). The data translate into a wide range of clinopyroxene crystallization depths that span from about 3 km to about 30 km for the 1999 eruption. The 2000 lavas, in turn, record more restricted crystallization depths of about 12 to 30 km. When employing the 50.2 Mg# nominal melt, clinopyroxene crystallization pressures range between 0.09 and 0.97 GPa for the 1999 lavas and between 0.36 and 0.91 GPa for the 2000 lavas (Fig. 4a,b). These pressure values translate into very similar depth ranges of between 3 and 35 km for the 1999 event and between 13 and 32 km for the 2000 eruption. Indeed, only limited variation in clinopyroxene derived pressure-depth results are produced when the full spectrum of equilibrium melt compositions is employed (see Discussion).

### Plagioclase-melt thermobarometry

**Figure 5:**
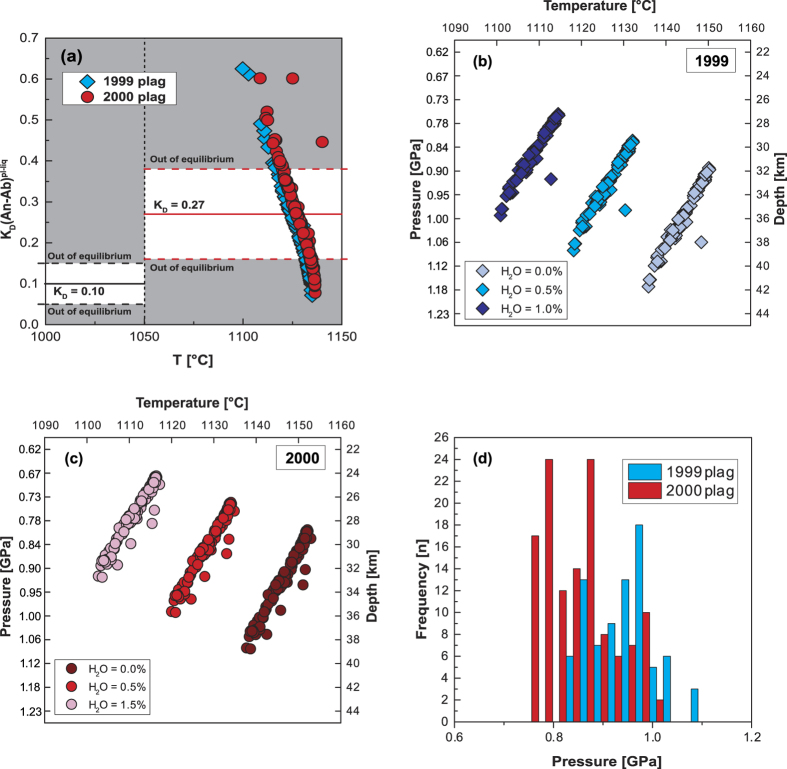
Plagioclase crystallization pressures for recent Mt. Cameroon lavas after Putirka^26^. (**a**) Equilibrium test for plagioclase crystals from the 1999 and 2000 eruptions employing a K_D[Ab-An]_ of 0.27 ± 0.11 at T ≥ 1050 °C (ref. 26) to define the equilibrium envelope. Water content in Mt. Cameroon lavas average around 0.6 wt% (ref. 11) and the effect of different H_2_O contents on plagioclase-melt thermobarometry is shown for the 1999 lavas in diagram (**b**) and for the 2000 lavas in (**c**), and is observed to be systematic in both cases (SEE = ±0.25 GPa, ±36 °C). (**d**) Plagioclase-melt thermobarometry after Putirka^26^ for plagioclase from the 1999 (blue) and 2000 (red) eruptions (at 0.5 wt% H_2_O) when coupled with equilibrium melts of Mg# 53.7 and Mg# 54.4, respectively (SEE = ±0.25 GPa).

For plagioclase, we employed the plagioclase-melt equilibrium thermobarometer of Putirka^26^. Suitable plagioclase mineral-melt pairs were selected for thermobarometry by applying the Kd_[Ab-An]_ equilibrium test, which is based on An-Ab exchange. This partition coefficient was shown to be constant with respect to magmatic P-H_2_O variations^26^. The whole rock data selected as nominal melt compositions when paired with the corresponding plagioclase are required to fall within the interval K_D[Ab-An]_ = 0.27 ± 0.11 for T ≥ 1050 °C and K_D[Ab-An]_ = 0.10 ± 0.05 for T < 1050 °C to satisfy equilibrium conditions. This test yielded ~48% of equilibrium plagioclase data points for the 1999 eruption and ~63% of equilibrium plagioclase data points for the 2000 eruption for predicted temperatures ≥1050 °C (Fig. 5a), which correspond to an anorthite range of 58 to 78 An mol% for these two eruptions.

Additionally, the plagioclase thermobarometry formulation of Putirka^26^ requires H_2_O input. Water content can be estimated from pressure and temperature obtained by thermobarometric modelling using the plagioclase hygrometer after Lange *et al*.^33^. However, this hygrometer is calibrated for pressures ≤ 0.30 GPa and is thus not applicable to the investigated Mt. Cameroon plagioclase. Data from Fitton^11^, however, provide an average of ~0.6 wt% volatiles from loss on ignition for recent whole rock samples from the continental sector of the Cameroon Volcanic Line, which is consistent with OIB and alkaline magmas that usually display low H_2_O contents (≤1 wt%)^34^. Therefore, a value of 0.5 wt% H_2_O appears a reasonable approximation for this study. Employing variable H_2_O input (lowering or increasing the H_2_O contents by e.g. 0.5 wt%) showed only small shifts in the overall thermobarometric results (Fig. 5b,c). The results of the model have a standard error of estimate (SEE) of ±0.25 GPa for pressure and ±36 °C for temperature^26^.

The derived plagioclase crystallization pressures range from 0.78 to 1.95 GPa for the 1999 lavas and from 0.71 to 1.51 GPa for the 2000 lavas (Fig. 5d). These values translate to plagioclase crystallization depths of between 29 and 39 km for the 1999 lavas and between 26 and 36 km for the 2000 lavas.

## Discussion

**Figure 6:**
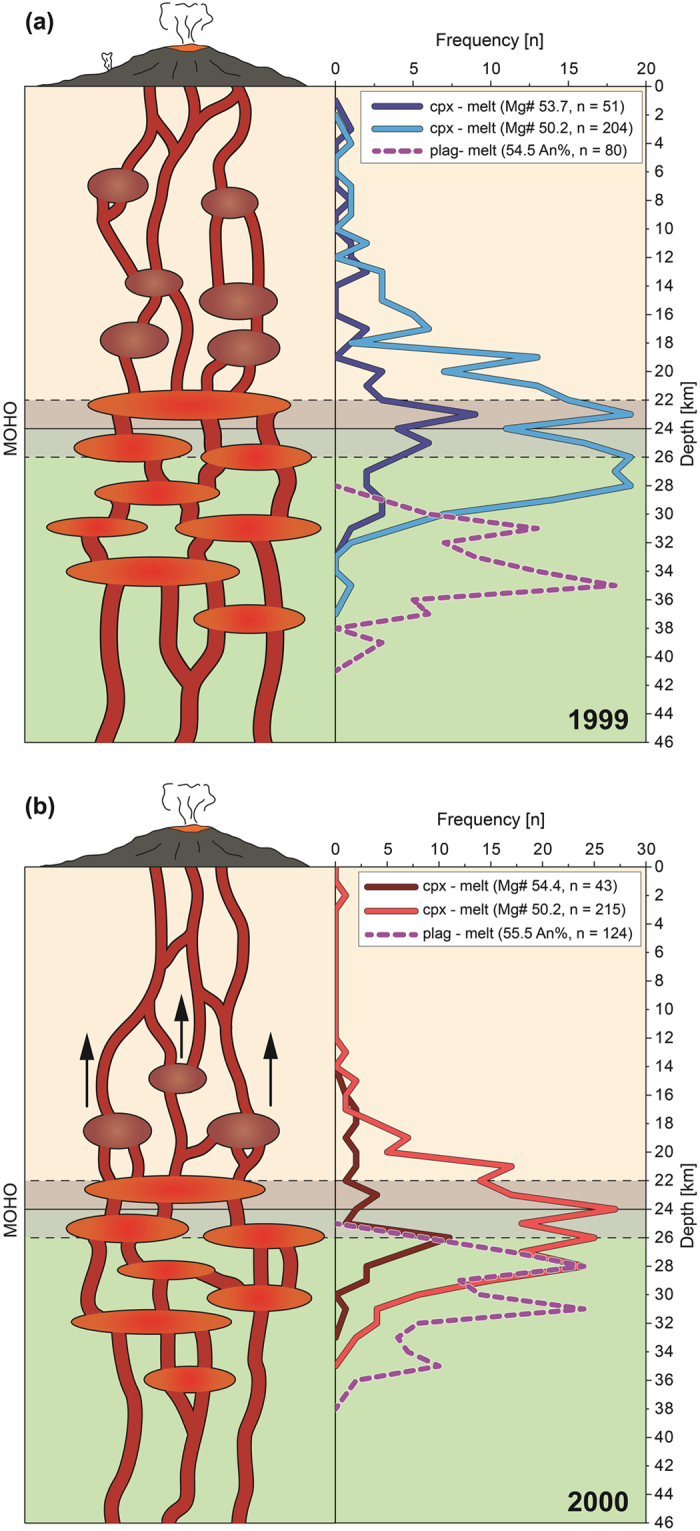
Schematic model of magma plumbing beneath Mt. Cameroon volcano. (**a**) The 1999 eruption and (**b**) the 2000 eruption are represented on the basis of our combined mineralogical and thermobarometry results. Both eruptions were predominantly fed from sub-Moho magma reservoirs, but shallower magma pockets existed prior to the 1999 eruption, which are not detected in the mineral data from the 2000 lavas. This observation implies that longer repose intervals, like prior to the 1999 eruption, may allow for ascent and evolution of small magma batches. These migrating pockets may also be a reason for seismic unrest and short explosive outbursts that occur in-between the major effusive events, e.g. in 1989 (ref. 13) and again in 2012 (ref. 15). Arrows in b) represent potential post-2000 magma migration. The SEEs are ±0.17 GPa and ±0.25 GPa for clinopyroxene-melt and plagioclase-melt thermobarometry, respectively.

The combined results of clinopyroxene-melt and plagioclase-melt thermobarometry indicate a multi-level plumbing system beneath Mt. Cameroon. Notably, plagioclase shows a major level of crystallization between 26 and 39 km depth (Fig. 6). This depth interval agrees with reported earthquake hypocentres at between 30 and 55 km depth beneath Mt. Cameroon prior to and during the 1999 and 2000 events, which were previously interpreted to signify magma migration at that depth^8^. Additionally, the early appearance of plagioclase before clinopyroxene is likely a function of high water pressures (pH_2_O) in mafic magmas, which will stabilise plagioclase crystallization at greater depth^4^.

In addition to the deep level of crystallization recorded by plagioclase, the dominant zone of clinopyroxene crystallization shows a range of between 20 and 28 km for the 1999 and 2000 eruptions (Fig. 6a,b), and thus also appears to have been sourced from a relatively deep storage level. These clinopyroxene data are consistent with previous seismic and petrochemical studies at Mt. Cameroon that suggested a feeder reservoir at ≥20 km for the 1999 and the 2000 events^8^,^13^. Moreover, the crust beneath Mt. Cameroon is comparatively thin^14^,^35^ and the Moho is located at 24 ± 2 km^35^, which overlaps with the depth of the main clinopyroxene crystallization level recorded by our data (Fig. 6a,b). We note that Moho and sub-Moho level crystallization appears to be a common feature in a variety of volcanic settings^19^,^36^,^37^,^38^.

Magmas that are directly derived from partial melting of the mantle are characterized by high whole rock MgO content (>8 wt%) and high Fo content of olivine^39^ (Fo_88–92_). However, the lavas from the recent Mt. Cameroon eruptions show lower whole rock MgO contents (~7.22 to ~7.29 wt%) and intermediate olivine compositions (Fo_67–86_). This observation implies that recent Mt. Cameroon magmas fractionated from primitive mantle melts after their generation. Our data highlight that this crystal fractionation process likely occurred below and around the Moho level (cf. refs 8 and 40). Therefore, the Moho beneath Mt. Cameroon appears to act as a density barrier at which magmas may stall and crystallize before ascending further to the surface.

Moreover, reverse zoning of plagioclase and clinopyroxene crystals from the 1999 and 2000 lavas points to mixing of two or more magma batches. This observation is consistent with the evidence from the 1982 Mt. Cameroon lavas that mixed with remnant magmas of the 1959 eruption^7^ (Fig. 2d). Comparing the 1999 and 2000 lava compositions with the 1959 and 1982 lava data on a Nb vs. K_2_O plot, a mixing trend between the 1999 and 2000 compositions and the 1982 compositions is indeed identified (Fig. 2d), and implies that remnant material from the 1982 eruption was incorporated into the 1999 and 2000 magmas. Mixing seems to have involved compositionally similar magmas in respect to major element compositions, as most of the clinopyroxene and plagioclase analyses are in equilibrium with nominal melts between Mg# 50.2 and 54.4. However, the presence of disequilibrium minerals, likely antecrysts, and the observed trace element trends suggest mixing between compositionally more distinct melts, such as perhaps some remnant and evolving 1982 magma batches.

Interestingly, thermobarometry of clinopyroxene from the 1999 eruption also records a sub-set of shallow crystallization pressures that are not observed in the 2000 eruptive products. We interpret these shallow mineral pressure results in the 1999 eruptives to represent pre-existing magma pockets at between 3 and 12 km depth and hence within the volcano’s upper plumbing system. These magma pockets were probably intersected by and amalgamated with the ascending 1999 magmas. The notable absence of significant clinopyroxene crystallization shallower than 12 km depth in the 2000 eruption products is then likely a function of the short repose time between the 1999 and 2000 events. The short repose intervals between the 1999 and 2000 events would only allow for limited ascent and differentiation of any remaining magma batches from the deep reservoir system. In contrast, the 17 year repose interval prior to the 1999 eruption would likely have facilitated migration, which led to independent chemical evolution of small volume magma batches in the mid- to upper crust. As the 2000 lavas do not contain such shallow grown crystals, we have to assume that the 1999 eruption had largely cleared the conduit system prior to the 2000 event (Fig. 6b). Notably, seismic activity occurred in-between the major eruptions as illustrated by seismicity down to 60 km depth associated with the phreatic explosions in 1989 and 2012 (refs 13 and 15), which may reflect migrating magma pockets during the inter-eruptive episodes (e.g. between 1982 and 1999, and since 2000). Repose time may therefore play a crucial role in the development of the shallower parts of the magma plumbing system beneath Mt. Cameroon and appears to allow ascent of small volume magma parcels that may produce shallow magma pockets and associated earthquakes and gas outbursts.

Future eruptions of Mt. Cameroon will probably follow the pattern displayed by these recent events. Recycling of shallow evolving magma pockets might therefore reoccur as implied by our results from the 1999 eruption. Such evolving shallow pockets may become gas-rich with sufficient repose time and when subjected to mafic recharge, they may become super-heated and re-mobilized to potentially trigger explosive events (e.g. refs 41, 42, 43, 44). We hence anticipate the possibility that mafic recharge from sub-Moho levels into an evolving shallow magma pocket could cause a scenario similar to the one observed during the 2010 eruption at Eyjafjallajökull volcano on Iceland. There, an almost 200 year old magma pocket associated with an eruption in 1824 was intersected and tapped^45^,^46^, which initiated the explosive phase of the 2010 Eyjafjallajökull events.

Monitoring of Mt. Cameroon volcano should therefore focus on magma migration from the sub-Moho storage system as patterns of shallowing earthquakes from this level are likely to be the precursors to major effusive eruptions at Mt. Cameroon in the future. Repose time and small-volume magma migration within the plumbing system of Mt. Cameroon appears to produce isolated and probably compositionally evolving shallower magma pockets. These may lead to sporadic and short-lived gas outbursts or minor eruptions and could, if intersected by ascending magma, cause temporary variations in eruptive style during larger future eruptive events.

## Methods

Weathered surfaces of lava samples were removed prior to jaw crushing and powdering in an agate mill at Uppsala University, Sweden. Major and trace element composition of bulk rock for 20 lava samples (15 for 1999 and five for 2000 lavas) were analysed at Acme Analytical Laboratories, Vancouver, Canada (http://acmelab.com) using LiBO_2_/Li_2_B_4_O_7_ fusion ICP-ES analysis for major elements and acid digestion ICP-MS analysis for trace elements. Method detection limits (MDLs) for major elements as oxides are 0.01 wt% except for Fe_2_O_3_ (0.04 wt%) and Cr_2_O_3_ (0.002 wt%). For trace elements, MDLs are in the range of 0.002 ppm to 20 ppm. Sample duplicates have reproducibilities of <0.10 wt% for most major elements and <10 ppm for trace elements. Accuracy of the methods was confirmed by internal reference materials (SO-18 for major elements, OREAS-24P and OREAS-45P for trace elements), with reproducibilities of <0.10 wt% for major elements and <5 ppm for trace elements. Major and trace element compositions of 1999 and 2000 lavas are presented in the supplementary information.

Clinopyroxene, plagioclase and olivine compositions were analysed in twelve samples (nine from the 1999 eruption and three from the 2000 eruption) by wavelength-dispersive spectrometry using a Cameca SX50 electron microprobe and a Jeol JXA8530F Hyperprobe Field Emission Electron Probe Microanalyser at the Centre for Experimental Mineralogy, Petrology and Geochemistry (CEMPEG), Uppsala University. Measurements were conducted under standard operation conditions of 20 kV accelerating voltage and 15 nA beam current for the Cameca SX50 electron microprobe (see Andersson^47^ for full analytical details) and 15 kV accelerating voltage and 10 nA beam current for the Jeol JXA8530F Hyperprobe^36^. The resulting data set is comprised of 972 spot analyses from 416 single crystals. This includes 527 analyses from 202 clinopyroxene crystals, 364 analyses from 145 plagioclase crystals, and 81 analyses from 69 olivine crystals (see Supplementary Information for full dataset).

## Additional Information

**How to cite this article**: Geiger, H. *et al*. Locating the depth of magma supply for volcanic eruptions, insights from Mt. Cameroon. *Sci. Rep*. **6**, 33629; doi: 10.1038/srep33629 (2016).

## Supplementary Material

Supplementary Information

## Figures and Tables

**Figure 1:**
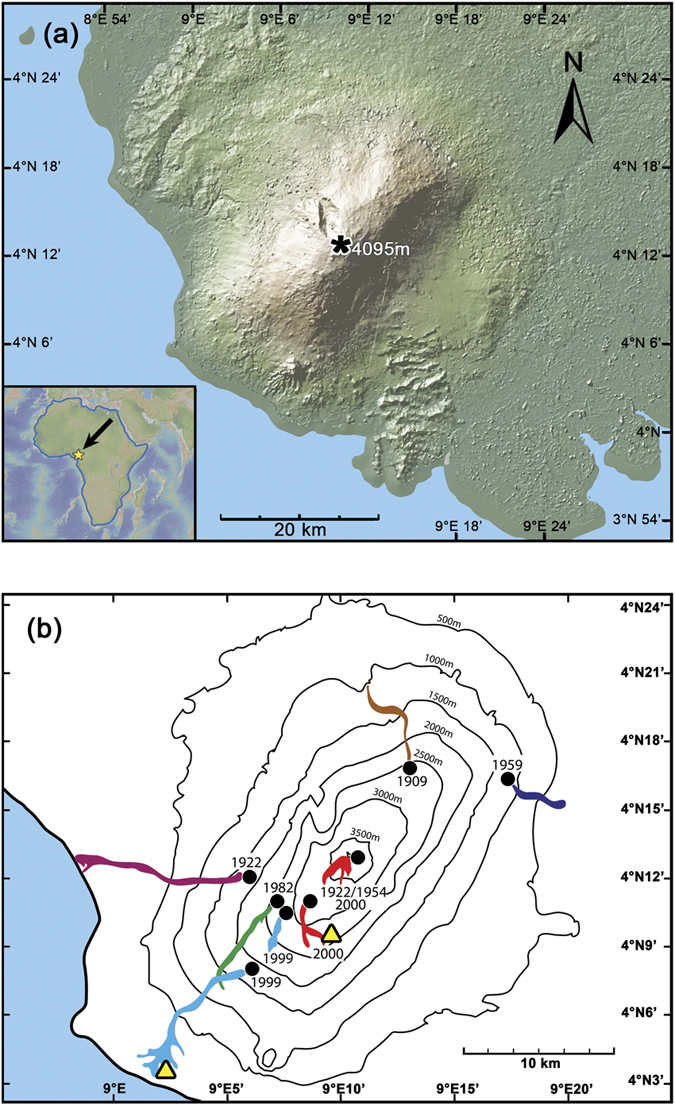
Location maps of Mt. Cameroon volcano and recent lava flows. (**a**) Digital elevation map of Mt. Cameroon volcano, West Africa (Source: GeoMapApp, http://www.geomapapp.org)^48^. The volcano’s summit is indicated by a black star. (**b**) Topographic map of Mt. Cameroon volcano with the lava flows of the last century highlighted in colour (after Suh *et al*.)^8^. Black dots mark the vents from which the lava flows were emitted. Sampling locations are marked by yellow triangles.
